# Targeting TNFR1-driven necroptosis in breast cancer

**DOI:** 10.17179/excli2025-8873

**Published:** 2025-12-01

**Authors:** Misbahuddin Rafeeq, Muhammad Afzal, Muhammad Shahid Nadeem, Alaa Hamed Habib, Hadeel A Alsufyani, Sami I. Alzarea, Omar Awad Alsaidan, Imran Kazmi

**Affiliations:** 1 Division of Basic Medical Sciences, College of Medicine, Dhofar University, Oman; 2 Department of Pharmaceutical Sciences, Pharmacy Program, Batterjee Medical College, P.O. Box 6231 Jeddah 21442, Saudi Arabia; 3 Department of Biochemistry, Faculty of Sciences, King Abdulaziz University, Jeddah 21589, Saudi Arabia; 4 Department of Physiology, Faculty of Medicine, King Abdulaziz University, Jeddah, 21589, Saudi Arabia; 5 Department of Clinical Physiology, Faculty of Medicine, King Abdulaziz University, Jeddah, 21589, Saudi Arabia; 6 Department of Pharmacology, College of Pharmacy, Jouf University, Aljouf, Sakaka 72341, Saudi Arabia; 7 Department of Pharmaceutics, College of Pharmacy, Jouf University, Sakaka, 72341, Al-Jouf, Saudi Arabia

**Keywords:** TNFR1, necroptosis, RIPK1-RIPK3-MLKL, breast cancer, tumor microenvironment, translational biomarkers

## Abstract

Tumor Necrosis Factor Receptor 1 (TNFR1) plays a crucial role in determining whether a breast cancer cell will survive, undergo natural cell death, or die through necroptosis. It influences these outcomes via pathways such as NF-kB, caspase-8, and the RIPK1-RIPK3-MLKL axis. TNFR1 activation causes epigenetic changes in DNA methylation, histone modification, and chromatin remodeling, which reprogram cellular responses to death signals. The direct and indirect epigenetic events leading to TNFR1-mediated cell death include DNMT enrolment, H3K4me3/H3K27ac changes, and microRNA-mediated controls. TNFR1 signaling regulates DNA methyltransferase activity and histone acetyltransferases while controlling epigenesis through metabolic reprogramming and non-coding RNA networks. The necroptotic execution pathway, triggered by pro-survival complex degradation and caspase-8 inhibition, forms the RIPK1-RIPK3 necrosome, phosphorylates MLKL, and releases damage-associated molecular patterns. TNF dual role of TNF signaling in tumor growth, necroptosis, and inflammatory remodeling presents therapeutic challenges. Biomarkers include TNFR1 expression, RIPK1/RIPK3 phosphorylation, MLKL localization, and epigenetic markers. Therapeutic combinations of epigenetic modulators, SMAC mimetics, RIPK1, and immune checkpoint inhibitors show promise in overcoming treatment resistance. Challenges in patient stratification, drug sequencing, and management of inflammatory toxicity require urgent solutions. This review provides a basis for clinical trials targeting the TNFR1-necroptosis pathway with biomarker-guided therapies and epigenetic strategies for breast cancer therapy.

See also the graphical abstract(Fig. 1).

## Introduction

Breast cancer is a leading cause of cancer-related mortality worldwide. The presence of considerable variability both among and within tumors makes it challenging to choose suitable treatment options and reduces the effectiveness of therapies (Korucu and Inandiklioglu, 2024[35], Smolarz et al., 2022[58]). While hormone receptor blockade, HER2-targeted therapies, and immunotherapy have shown substantial improvements in their outcomes, resistance and metastatic progression continue to impact the results in molecular subtypes (Derakhshan and Reis-Filho, 2022[14], Schandiz et al., 2023[57]). Factors such as inflammatory signaling pathways and other elements within the tumor microenvironment play a crucial role in determining disease progression and response to treatment (Kulothungan et al., 2024[36], Sathishkumar et al., 2022[54]).

The tumor necrosis factor receptor 1 (TNFR1) plays a crucial role in determining whether a cell in breast cancer will survive, undergo apoptosis, or experience necrosis. The interaction of the TNF-α ligand leads to the stabilization of receptor-proximal Complex I, which includes TRADD, TRAF2, and cIAPs, thereby promoting NF-kB-dependent survival signaling (Chen et al., 2019[7], Dondelinger et al., 2016[15]). When ubiquitin scaffolds are lost or caspase-8 is inhibited, the pathway shifts towards cytosolic death complexes, triggering necroptosis through the RIPK1-RIPK3-MLKL axis. TNFR1 signaling also leads to extensive epigenetic changes that alter the cellular response to death (Reilly et al., 2022[52], Vince et al., 2009[64]). TNF-α has been shown to recruit DNA methyltransferases and enzymes that modify histones to promoters, establishing chromatin configurations that favor survival. This includes mechanisms such as the enhancement of the H3K4me3 state at pro-survival sites and the action of chromatin-remodeling complexes on NF-KB targets (Moriwaki and Chan, 2016[49], Zhang et al., 2024[77]). Indirect control mechanisms are established when TNF-α signaling impacts microRNA networks that regulate gene activity, modifies the levels of long non-coding RNAs, and influences the metabolic cofactors that interact with enzymes involved in chromatin modification.

Phosphorylation of MLKL leads to necroptosis, causing the cell membrane to become permeable and releasing damage-associated molecular patterns (DAMPs) that alter the tumor microenvironment. These DAMPs have the potential to trigger antitumor immune responses by activating dendritic cells and T-cells (Amaravadi et al., 2015[1], Guo et al., 2025[22]). However, the same immunogenic signals from tumors can also facilitate tumor progression by reorganizing the stroma and promoting angiogenesis, highlighting the inherent dual nature of TNF-α-mediated necroptosis. Therapeutic approaches targeting TNF-α signaling encounter anticipated challenges, including the dual role of this pathway in both promoting and inhibiting tumor growth, its dependence on specific contexts, and the established risk of metastasis resulting from uncontrolled inflammation. The complexity of these issues is increased by the varied epigenetic targets found in different breast cancer subtypes, highlighting the need for customized strategies to ensure successful clinical implementation (Grönholm et al., 2021[20], Yao et al., 2025[73]).

**Figure 2:**
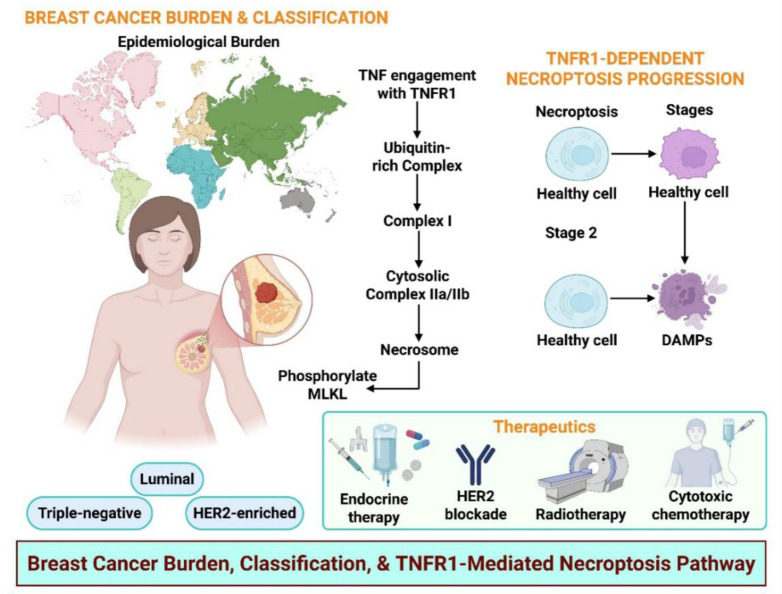
TNFR1 as a fate switch in breast cancer: NF-κB survival vs apoptosis and RIPK1-RIPK3-MLKL necroptosis

This review examines the epigenetic processes that control TNFR1-triggered cell death, the potential for therapeutic disruption of both direct and indirect epigenetic points, and the challenges faced in applying TNF-α pathway interventions in practice. By integrating molecular pathways with biomarker development and therapeutic strategies, we propose a model of rational combinations that incorporates epigenetic modulators to overcome resistance and achieve sustained tumor control (Figure 2(Fig. 2)).

## TNFR1 Signaling as a Molecular Switch in Determining Cell Fate

### Structural features and ligand-binding dynamics of TNFR1

TNFR1 is a type I transmembrane receptor with four cysteine-rich domains (CRD1-CRD4): CRD1 stabilizes pre-assembly, CRD2-CRD3 contains the TNF interface, and CRD4 positions binding near the membrane to couple with intracellular death domain signaling. This signals NF-κB-mediated survival, caspase-8-mediated apoptosis, or RIPK1 RIPK3, and mixed lineage kinase domain-like protein (MLKL)-mediated studies (He et al., 2021[24], Rivas et al., 2008[53]) show that pre-assembled TNFR1 arrays enhance responsiveness by reducing the energetic costs of trimer interactions. The ligand form is a key determinant: membrane-bound TNF (tmTNF) forms high-affinity interactions that maintain multivalent TNFR1 interactions, whereas soluble TNF (sTNF) promotes transient interactions. El Yazidi-Belkoura et al. showed that tomatoes have longer receptor residency (El Yazidi-Belkoura et al., 2003[16]), and Branschädel et al. correlated signal strength with ligand concentration and receptor pre-assembly (Branschädel et al., 2010[5]). This aligns with receptor-proximal findings: stabilized ubiquitin-rich Complex I enables NF-kB-dependent survival; reduced ubiquitin scaffolds or restricted caspase-8 leads to cytosolic Complex II variants causing apoptosis or, with RIPK1 kinase, necrosome assembly, and necrosis (Cruceriu et al., 2024[10], Xu et al., 2006[69]).

These ligand effects are modulated by the cell surface, resolving model discrepancies. CRD2-CRD3 accessibility and trimer formation are modulated by N-glycosylation and receptor density. Division into cholesterol-/sphingolipid-rich microdomains facilitates TNFR1 and adaptor protein aggregation to enhance Complex I in pro-survival environments or promote death signaling when checkpoints are dysfunctional. Soluble TNFR1 from proteolytic shedding buffers local TNF and reconfigures gradients, whereas TNFR2 crosstalk modulates ligand and adaptor availability. Studies have linked CRD-directed receptor sorting and ligand shape to fate selection in breast cancer.

These membrane logics are connected to epigenetic states: NF-kB occupancy by Complex I supports chromatin-modifying enzymes and pro-survival states, while scaffold collapse with defective caspase-8 enables necroptosis through chromatin reprogramming. TNFR1 fate and downstream epigenetic effects are programmed by structural determinants, ligand form, and membrane (Cruceriu et al., 2024[10]). Future studies should quantify fMnTNF versus sTNF interactions and Complex I residency in physiological membranes. Combining receptor biophysics with adaptor ubiquitin architecture and chromatin profiling will enable the rational selection of combinations directing TNFR1 to therapeutic death programs.

### Balancing pro-survival and pro-death signaling outputs

Receptor-proximal checkpoints regulate TNFR1 signaling to determine whether NF-κB-mediated cell survival will persist or switch to apoptosis or necroptosis. After ligation, a membrane-bound Complex I forms around TNFR1, including TRADD, RIPK1, TRAF2/5, cIAP1/2, and LUBAC, stabilized by the TAB/TAK1 and IKK complexes to maintain survival transcription. This survival bias aligns with clinical results: higher TNF-alpha correlates with higher stage and worse prognosis (Hu et al., 2024[26]), and TNFR1 deficiency reduces tumor growth and metastasis (Yang et al., 2017[70]). With maintained ubiquitin platforms and TAK1/IKK signaling, pro-survival transcription occurs, and caspase activation is inhibited (He et al., 2021[24]).

Deubiquitinases or cIAPs depletion destabilizes Complex I, enabling cytosolic death-inducing assemblies. When caspase-8 is balanced with cFLIP-L, apoptosis occurs through BID activation and mitochondrial permeabilization (Xu, 2006[69]). With defective caspase-8 activity or TAK1/IKK, RIPK1 kinase interacts with RIPK3 and MLKL for necroptosis, supported by evidence that necrostatin-1 suppresses cell death and TNF stimulates MLKL phosphorylation (Lin et al., 2004[40], Zhang et al., 2018[79]). High RIPK1/RIPK3/MLKL levels correlate with decreased viability, necessitating necroptotic axis profiling (Liu et al., 2016[41]). Membrane-anchored TNF is demonstrably more effective in promoting high-avidity clustering, which is a crucial element for successful cellular interactions. In contrast, sTNF leads to shorter and less consequential interactions. This distinction highlights the essential role of membrane-anchored TNF in augmenting cellular communication and functions (Branschädel et al., 2010[5]). Signal strength is related to ligand concentration and TNFR1 pre-assembly (Branschädel et al., 2010[5]). Cholesterol-rich microdomains enhance Complex I survival, whereas weak scaffolds favor death complexes (He et al., 2021[24], Rivas et al., 2008[53]). TNFR2 inhibition restrains cell proliferation and, when combined with PD-L1 inhibition, increases CD8+ T cell (Fu et al., 2021[19]). Under certain conditions, TRAIL co-engagement with tmTNF can increase chemoresistance (Xu et al., 2006[69], Yoon et al., 2016[75]). 

NF-κB activation mediated by TRADD stimulates growth and can be negated by TRADD knockdown, consistent with survival in Complex I stabilization (Cai et al., 2017[6]). Heregulin suppresses TNFR1NF-κB and prepares cells for apoptosis, providing an advantage for cell death (Moatti and Cohen, 2021[46], Sprowl et al., 2012[60]). These data suggest that equivalent TNF exposure has different effects in breast cancer: ubiquitin architecture, caspase-8/cFLIP balance, kinase competency for RIPK1 necroptosis, ligand form, and receptor microdomain residency combine to favor survival, apoptosis, or necroptosis.

These proximity receptor decisions create epigenetic fate-reinforcing decisions. Stabilized Complex I maintains NF-Kb occupancy at enhancers and promoters, recruiting histone acetyltransferases, SWI SNF remodelers, and methyltransferases to establish pro-survival chromatin. Complex II or necrosome formation coincides with the redistribution of DNA/histone enzymes and reprogramming of non-coding RNA and metabolic cofactors, enabling efficient apoptosis or necroptosis. Future efforts should combine ubiquitin-scaffold integrity and RIPK1 activity readouts with chromatin profiling to identify predictive biomarkers. Modulating TNFR1 gating through rational combinations while monitoring the epigenetic state will guide results towards therapeutic cell death with reduced pro-tumor inflammation.

## Molecular Framework of TNFR1-Driven Necroptotic Signaling

### Activation and regulation of RIPK1-RIPK3-MLKL axis

Necroptotic execution downstream of TNFR1 occurs when pro-survival scaffolds degrade and caspase-8 is inhibited, allowing RIPK1 to form a necrosome with RIPK3, which phosphorylates MLKL. Phosphorylated MLKL oligomers disrupt membrane integrity, leading to cell death and DAMP release. This process depends on adaptor loading, caspase competence, ligand formation, and membrane organization. High RIPK1 levels correlate with enhanced necroptosis, TNF pathway upregulation, and poor survival (Yoon et al., 2016[75]). RIPK3 knockdown reduces MLKL phosphorylation, necroptotic death, HMGB1/ATP release, and CD8⁺ T cell infiltration, whereas MLKL activation promotes dendritic cell maturation (Yang et al., 2016[71]). Phosphorylated MLKL rapidly translocates to the nucleus, and blocking nuclear import reduces necroptosis (Snyder et al., 2019[59], Yoon et al., 2016[75]). In triple-negative models, AQP1 silencing reduces growth, whereas RIPK1 knockdown rescues proliferation (Hou et al., 2019[25]). RIPK1/RIPK3 activation increases CD8⁺ cells and IFN-γ, reduces tumors, and improves survival, especially with PD-1 blockade (Wang et al., 2024[65]). RIPK1 inhibitors suppress necroptosis and improve survival (Cai et al., 2017[6]). A seven-gene necroptosis classifier was used to stratify risk and PD-L1 levels (Liu et al., 2016[41]). These studies show how receptor-proximal events lead to necrosome formation, demonstrate context-dependent immunogenic effects, and support strategies using RIPK1 inhibition or checkpoint-partnered necroptosis induction.

### Interplay between apoptosis, necroptosis, and inflammatory responses

**Figure 3:**
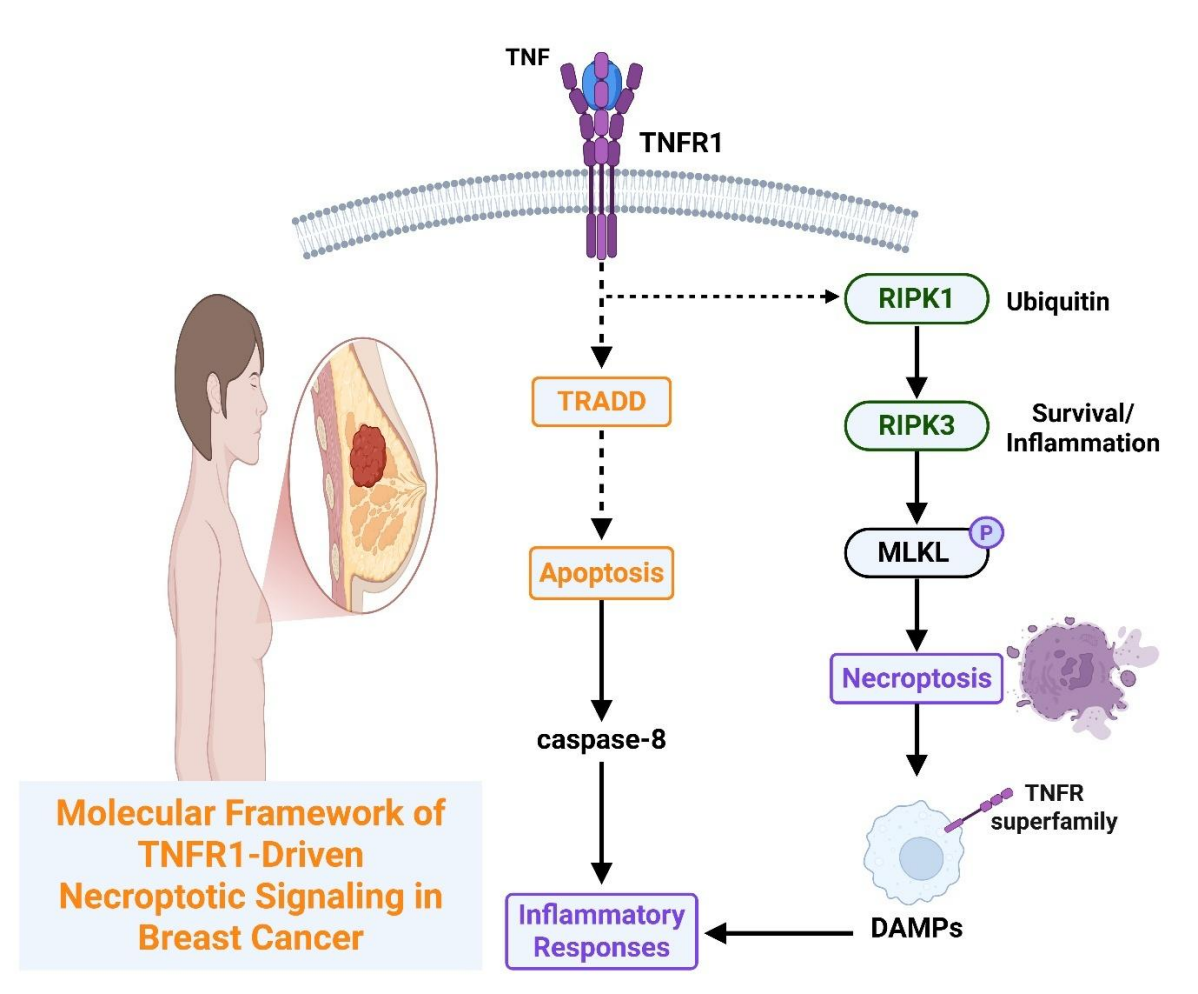
TNFR1 complex architecture and checkpoint control (complex I → IIa/IIb/IIc) governing apoptosis versus necroptosis

The downstream fate of TNFR1 depends on the inflammatory tone, receptor-proximal complex assembly, and caspase/RIP checkpoints. TNF-α establishes a pro-survival baseline by enhancing NF-κB activity, proliferation, and in vivo growth, positioning inflammation as a driver of tumor fitness (Zou et al., 2020[82]). Increased TRADD recruitment enhances NF-κB and suppresses apoptosis, whereas reducing TRADD restores caspase-3 activity (Cai et al., 2017[6]). TNFR1 activation increases proliferation, whereas its silencing enhances caspase-3 and tumor apoptosis, indicating that the balance between Complex I signaling and death-complex formation is modifiable (Xu et al., 2006[69]). When caspase-8 is restrained and RIPK1 is competent, signaling shifts to regulated necrosis. In breast cancer, RIPK1/RIPK3 upregulation and MLKL activation are correlated with reduced viability and poor prognosis (Moatti and Cohen, 2021[46], Sprowl et al., 2012[60]). TNF-α plus TRAIL increases apoptosis, enhances doxorubicin cytotoxicity, and raises caspase-8 levels (Liu et al., 2016[41]). Additionally, heregulin suppresses TNFR1-NF-κB signaling and increases TNF-α-induced apoptosis (Moatti and Cohen, 2021[46], Sprowl et al., 2012[60]). These studies show that inflammatory inputs bias towards survival, while rational combinations can tip the balance towards antitumor death (Figure 3(Fig. 3); Table 1(Tab. 1); References in Table 1: Bai et al., 2024[3]; Bilecova-Rabajdova et al., 2014[4]; Cai et al., 2017[6]; Chinnaiyan et al., 2000[8]; Chun et al., 2021[9]; Davey et al., 2021[12]; El Yazidi-Belkoura et al., 2003[16]; Eteshola et al., 2021[17]; Fu et al., 2018[18]; Fu et al., 2021[19]; Guo and Yuan, 2020[21]; He et al., 2021[24]; Hou et al., 2019[25]; Hussain et al., 2017[27]; Katanov et al., 2015[29]; Ke et al., 2023[30]; Khalili-Tanha and Moghbeli, 2021[31]; Kochumon et al., 2021[34]; Lafont et al., 2017[37]; Li et al., 2020[39]; Li et al., 2023[38]; Lin et al., 2004[40]; Moatti and Cohen, 2021[46]; Moerke et al., 2019[47]; Montinaro et al., 2022[48]; Nicolè et al., 2022[50]; Peterson et al., 2017[51]; Scarpellini et al., 2023[56]; Sprowl et al., 2012[60]; Van Berckelaer et al., 2024[62]; Vanden Berghe et al., 2016[63]; Wang et al., 2024[65]; West, 2019[66]; Wu and Zhou, 2010[68]; Xu et al., 2006[69]; Yang et al., 2016[71]; Yao et al., 2025[72]; Yin et al., 2021[74]; Yoon et al., 2016[75]; Yu et al., 2022[76]; Zhang et al., 2018[79]; Zhang et al., 2022[78]; Zheng et al., 2025[81]).

## Regulatory Networks Modulating TNFR1-Induced Necrosis

### Extracellular cytokines, chemokines, and stress stimuli

The impact of TNFR1 signals is shaped by external cytokines, chemokines, and stromal signals, which guide breast cancer cells towards either survival or programmed cell death (PCD). TNF-α can promote survival in the tumor epithelium. Cai et al. demonstrated that exposure leads to increased proliferation and invasion, along with a notable rise in antiapoptotic mechanisms such as Bcl-2 and cyclin D1, while significantly reducing apoptotic commitment. This epithelial tendency is further reinforced by the transcriptional regulation of the inflammatory environment (Cai et al., 2017[6]). Meškytė et al. found that inhibiting ETV7 transcription triggers the expression of IL-6 and TNF-A, and that ETV7 inhibition reduced these cytokines, indicating that tumor-intrinsic programs might enhance or diminish the ligands that bind to TNFR1 (Meškytė et al., 2023[44]).

The microenvironment enhances these epithelial signals. Katanov et al. (2015[29]) found that after cancer development, fibroblasts release IL-6, IL-8, and CCL2, and this stromal secretion aids in the growth and migration of tumor cells, which is linked to the increased activity of fibroblasts(Katanov et al., 2015[29]). At the receptor level, He et al. observed that tumors exhibit increased TNFR1 expression compared to the corresponding normal tissue, along with elevated NF-kB activity, which leads to greater proliferative behavior. This places receptor density and transcriptional output on a shared axis that supports survival signaling in the presence of cytokines (He et al., 2021[24]). Downstream pathways present a clinical risk at a later stage. Elevated levels of RIPK1, RIPK3, and MLKL are associated with a higher likelihood of recurrence and extensive overexpression of the necroptotic machinery in tumors (Liu et al., 2016[41]). This suggests that even when upstream signals favor survival, inflammatory death mechanisms are ready and can be triggered if checkpoints fail. This scenario can be altered by counter-regulatory growth factors. Heregulin suppresses TNFR1/NF-KB signaling, activates caspase-3, reduces viability in the presence of TNF-α, and shifts the outcome towards apoptosis. Consequently, Zou demonstrated that the shift towards apoptosis can be pharmacologically influenced by a receptor-proximal survival bias (Zou et al., 2020[82]). In studies of diseases influenced by survival bias, it is crucial to incorporate cytokine profiles, stromal signatures, and TNFR1 levels for effective patient stratification. The development of therapeutic interventions should focus on targeting the inflammatory feedback loop induced by cancer-associated fibroblasts (CAFs) and the effectors RIPK1/3MLKL, with the aim of mitigating proliferative signaling and necro-inflammatory remodeling. HRG-like modulators that regulate TNFR1/NF-kB and restore caspase-dependent execution should be considered as sensitizers in biomarker-guided combination therapies.

### Intracellular adaptors, inhibitors, and post-translational modifications

Intracellular adaptors like TRADD and FADD, inhibitory nodes from TNFR2, and post-translational modifications including RIPK1 ubiquitylation and RIPK1/MLKL phosphorylation determine whether TNFR1 signaling promotes survival, apoptosis, or necrosis. Ubiquitin editing stabilizes Complex I, whereas caspase-8 enables necroptosis transition. TNFR2 interacts with this network by competing with the ligand and adaptor pools and modifying the residency threshold of Complex I. In breast tumors, TNFR2 levels are elevated compared to those in normal tissue, which is associated with increased NF-KB production and promotes growth; its inhibition reduces these signals, while TNFR1 is associated with an apoptotic phenotype (He et al., 2021[24]). When tumor cells are exposed to TNF-alpha, the survival pathway is enhanced by boosting NF-kB targets and pro-growth cytokines, leading to accelerated in vivo expansion, indicating that receptor-proximal bias is prioritized over transcriptional reinforcement (Cai et al., 2017[6]). The concept of natural-product modulation emphasizes the same gatekeeping principle: Erioctyol reduces TNFR1 levels, decreases TRADD/FADD recruitment, suppresses NF-kB, and restores caspase-3 activity, thereby steering the outcome towards apoptosis in the presence of TNF-α (Debnath et al., 2022[13]).

Transcriptional programs also play a role in influence this pathway. When ETV7 is inhibited, it triggers the activation of interferon-stimulated genes, reduces STAT1 activation, and accelerates tumor growth. This suggests that the breakdown of an antiviral-like tone does not limit the growth of tumors responsive to TNF-alpha (Meškytė et al., 2023[44]). Microenvironmental cytokines contribute to this effect; cancer-associated fibroblasts secrete IL-1 2-linked chemokines and interleukins, which enhance proliferation and motility, effects that are mitigated when NF-kB is inhibited (Katanov et al., 2015[29]). High levels of RIPK1, RIPK3, and activated MLKL are associated with more severe disease. Inhibition of RIPK1/3 via genetic or pharmacological means can halt cell proliferation, metastasis, and tumor progression. Consequently, the necroptotic module serves as a valuable target for treatment and prognosis (Liu et al., 2016[41]). Counter-regulatory growth mechanisms have the potential to counteract these survival signals. Heregulin inhibits TNFR1/NF-KB, activates caspase-3, reduces cell viability, and diminishes tumor size in TNF-a environments. This indicates its capability to reverse survival signals by utilizing natural contextual cues (Zou et al., 2020[82]). System-level analyses have cautioned that alterations in the TNF axis can influence drug exposure and tumor cell apoptosis. This suggests that drug behavior and pathway regulation are interconnected and require careful equilibrium (Sprowl et al., 2012[60]).

To mitigate deleterious inflammation without disrupting the TNFR2-adaptor interaction, it is advisable to target TNFR2 by modifying its survival structure. This approach should be complemented by the regulation of the RIPK1/RIPK3-MLKL pathway to curtail harmful inflammation. Monitoring drug concentrations is crucial for ensuring a consistent and beneficial therapeutic response. This can be accomplished by preparing the axis for the TNF-adaptor interaction and maintaining therapeutic levels.

### Epigenetic mechanisms in TNFR1-mediated cell death

#### Direct epigenetic mechanisms

TNF-α can influence DNA methylation by attracting DNA methyltransferases (DNMTs) to the promoters of genes. In breast cancer cells, TNF-α is known to increase DNMT1 levels by approximately 2.4 times, leading to hypermethylation of tumor suppressor genes such as p16 and BRCA1, which shifts chromatin towards survival pathways (Mirza et al., 2013[45]). This methylation reduces the transcriptional accessibility of death receptor promoters. These effects were observed within one or two days of exposure. In addition, TNFR1 regulates TET enzymes. TNF stimulation reportedly reduces TET2 activity by approximately 40 %, hindering the demethylation of BAX and PUMA promoters and creating a sustained antiapoptotic expression state that persists beyond the initial stimulus (Zhang et al., 2022[80]).

**Figure 4:**
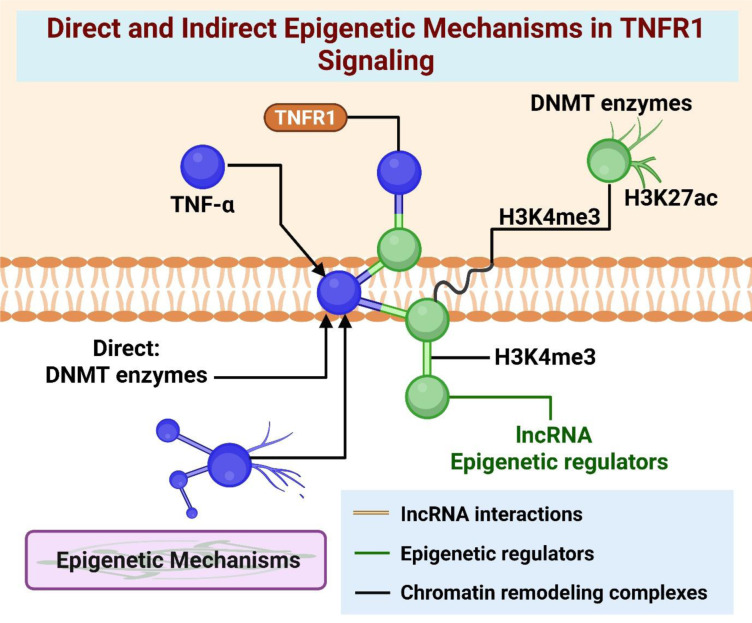
"Direct and Indirect Epigenetic Mechanisms in TNFR1 Signaling" - Schematic showing DNA methylation, histone modifications, and chromatin remodeling directly induced by TNF-α, alongside indirect effects through miRNA and lncRNA networks.

TNF-α uses transcriptional complexes containing NF-κB to attract histone-modifying enzymes to loci responsive to TNFR1. Modifications in chromatin that promote survival, such as increased H3K4me3 levels at BCL2 and XIAP (facilitated by MLL1) and elevated H3K27ac levels due to p300/CBP activity, enhance NF-κB-dependent transcription (Jang et al., 2021[28]). Conversely, when LSD1 binds to pro-apoptotic promoters, it reduces H3K4me3 levels and inhibits cell death. Marks that favor survival appear within minutes, whereas repressive marks on death-related genes accumulate over several hours, reflecting a coordinated, epigenetic response. TNFR1 signaling recruits ATP-dependent remodeling, particularly involving SWI/SNF complexes. It has been observed that TNF-α enhances BRG1 presence at NF-kB target promoters by increasing nucleosome accessibility and preserving transcriptional memory from prior exposure, thereby sustaining antiapoptotic conditions (Kim et al., 2018[33]) (Figure 4(Fig. 4)).

#### Indirect epigenetic mechanisms

The TNF-α signaling pathway intersects with the miRNA networks that target epigenetic enzymes. Members of the miR-29 family are reduced by approximately 60 %, allowing DNMT3A/3B to be released, which facilitates de novo methylation at tumor-suppressor sites (Amodio et al., 2015[2]). Additionally, TNF-α decreases miR-21 levels, which in turn stimulates UTX targeting to reduce H3K27me3 demethylation, a process linked to the suppression of pro-apoptotic genes expression. TNF-α can enhance lncRNAs that create epigenetic complexes. HOTAIR reportedly increases by approximately 4.2 times and attracts PRC2 to silence death receptor genes via H3K27me3. MALAT1 is believed to capture LSD1-complexes, thereby inhibiting the activation of pro-apoptotic chromatin (Saviana et al., 2023[55]). TNF-α can restrict TET and Jumonji demethylases that require this cofactor, leading to increased DNA hypermethylation and repressive histone changes. This process ultimately fosters a state that supports survival by enhancing glycolytic activity and reducing the α-ketoglutarate levels (Das et al., 2013[11]).

**Figure 5:**
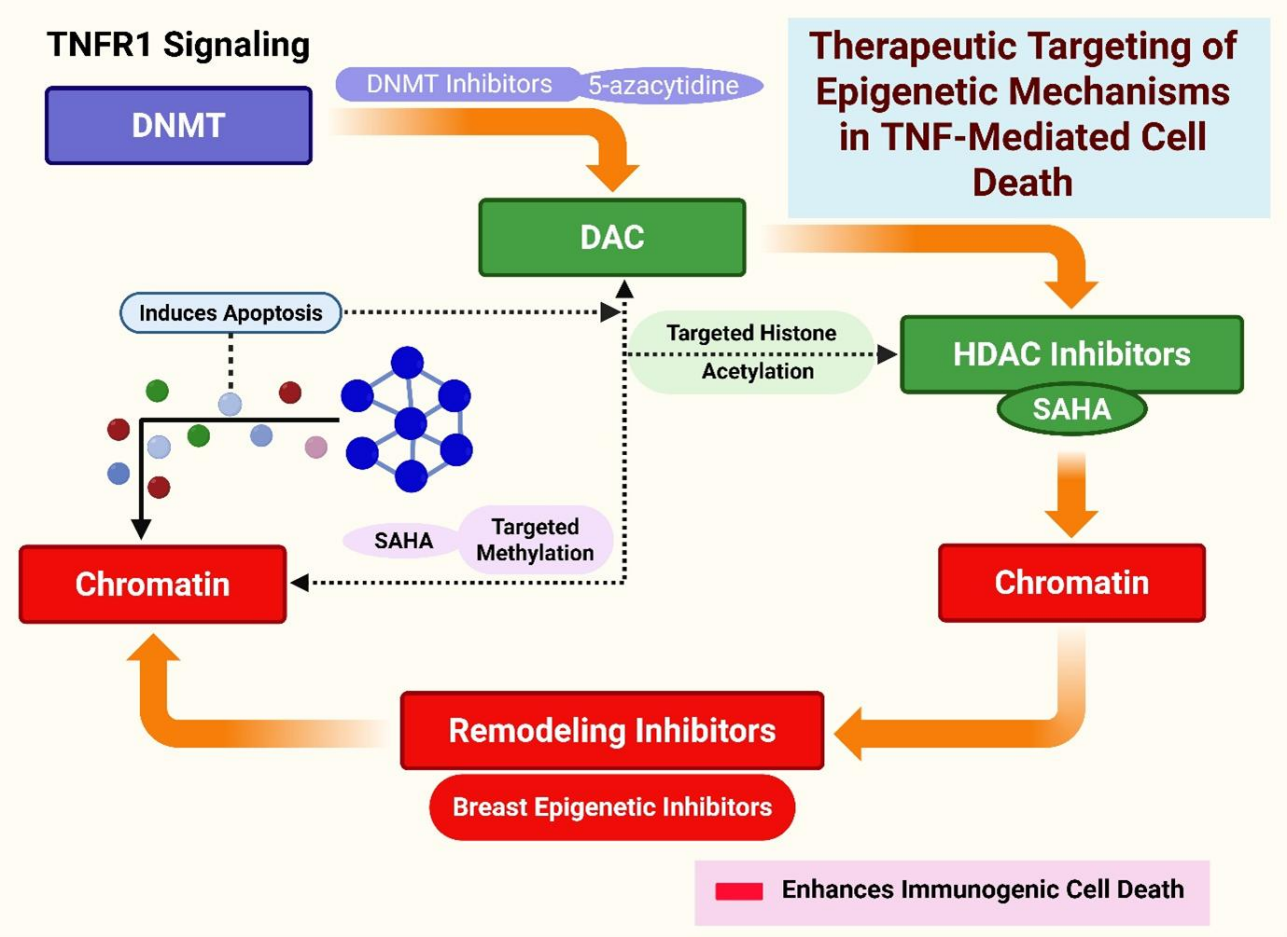
"Therapeutic Targeting of Epigenetic Mechanisms in TNF-Mediated Cell Death" - Overview of epigenetic inhibitors (DNMT, HDAC, chromatin remodeling inhibitors) and their points of intervention in the TNFR1 pathway for breast cancer treatment.

Indirect effects may be amplified through interactions with other inflammatory pathways. It has been noted that TNF-α activates STAT3, which in turn significantly increases EZH2 and DNMT1 levels, by approximately threefold and twofold, respectively, and promotes a pro-inflammatory, pro-survival transcription feedback loop (Wu et al., 2017 67[67]) (Figure 5(Fig. 5)).

#### Clinical implications and therapeutic targeting

The dual role of epigenetic regulation in TNFR1 signaling offers promising therapeutic opportunities. Hyper-methylation associated with TNF-α can be counteracted by DNMT inhibitors, such as 5-azacytidine, and when combined with TNF-α, these inhibitors have been found to induce higher levels of apoptosis than when used alone (Sullivan et al., 2007[61]). Additionally, HDAC inhibitors, such as SAHA, can reverse deacetylation at death-gene promoters caused by TNF-α, thereby reopening pro-apoptotic chromatin. When used alongside TRAIL or other death receptor agonists, these combinations can effectively counteract survival signaling across various models (Kim, 2025[32]). Changes in methylation at TNF-α-responsive promoters are linked to treatment responses, with hypermethylated tumors showing reduced sensitivity to standard treatments. Histone-mark profiles can help identify patients who are most likely to benefit from epigenetic therapies that target the TNF-α pathway (Luo et al., 2024[43]).

#### Limitations and future directions

The dual role of TNF-α, which can either promote or suppress tumors depending on the context, complicates systemic interventions. Broad TNF-α blockade might hinder antitumor immunity, whereas stimulation of the pathway could trigger metastatic processes by increasing inflammation. Timing is crucial, as epigenetic processes operate on a different timeline than receptor signaling. The specific heterogeneity of epigenetic landscapes suggests that personalized biomarker-guided approaches are necessary. Future efforts should focus on identifying multi-level combinations, such as DNMT, HDAC, and chromatin-remodeling targets, and validating their selection based on pathway-linked biomarkers. Additionally, pharmacodynamic monitoring is required to optimize the sequence and dosage. Single-cell epigenomics, which integrates functional readouts, will be vital for addressing the heterogeneity that leads to resistance and progression.

## Necro-inflammatory Consequences of TNFR1 Activation in Breast Cancer Microenvironment

### Damage-Associated Molecular Patterns (DAMPs) release and immune cell infiltration

DAMPs released from stressed or dying breast cancer cells enhance inflammation and influence immune cell trafficking, whereas cytokines modulate whether this environment inhibits or promotes disease progression. TNFR1 integrates DAMP signals with NF-κB/STAT outputs to influence cell survival or death. TNF-α initiates a pro-tumor program in breast cancer cells, increasing proliferation and IL-6/IL-8 and VEGF production in these cells. TNFR1 blockade reverses over half of these effects, establishing it as an inflammatory driver (Cai et al., 2017[6]). In inflammatory breast cancer, XIAP overexpression compared to non-inflammatory breast cancer is associated with elevated TNF-α/IL-6 levels and poorer survival, linking apoptosis resistance to an aggressive phenotype (Van Berckelaer et al., 2024[62]). A TNFR1-targeted 18F PET probe showed higher uptake in inflamed tissue than in controls, with reduced signal by blocking, supporting non-invasive quantification of inflammation and patient monitoring (Fu et al., 2018[18]). Tumor-derived DAMPs activate myeloid cells, increasing macrophage TNF-α and IL-6 levels and CD86⁺ fractions; this activation enhances tumor cell proliferation and migration, evidencing a DAMP-macrophage loop that perpetuates necro-inflammation (Eteshola et al., 2021[17]). These studies outline a sequence from cytokine amplification through antiapoptotic buffering to measurable pathway activity and immune remodeling. Three strategies have emerged: combining TNFR1 antagonism with DAMP signaling mitigation, using TNFR1 PET for patient monitoring, and disrupting macrophage reinforcement to transform necro-inflammation into productive immune control. These findings address how DAMP biology and TNFR1 signaling influence the immune context of breast cancer.

### Interaction with tumor-associated stromal and immune components

Tumor-stroma interactions regulate the necro-inflammatory environment through cytokines from mesenchymal and immune cells via tumor-intrinsic pathways, with TNFR1 playing a central role. Katanov et al. showed that mesenchymal stromal cells increased IL-6 by 2.5-fold and TNF-α by 1.8-fold, decreased IFN-γ by 40 %, induced M2-like macrophages, and accelerated xenograft growth, creating an immunosuppressive niche (Katanov et al., 2015[29]). Cai et al. demonstrated that TNF-α enhanced tumor fitness, increasing viability by 40 %, reducing apoptosis by 25 %, accelerating wound closure, and elevating NF-κB and cyclin D1 levels, linking stromal cytokines to tumor cell proliferation (Cai et al., 2017[6]). Van Berckelaer et al. (2024[62]) found that XIAP overexpression was observed in 76 % of inflammatory breast cancers versus 42 % of non-inflammatory cases, with reduced survival and increased NF-κB activity (Cai et al., 2017[6]). Debnath et al. showed that eriodictyol reduced TRADD/FADD complex formation, decreased NF-κB activity, reduced TNF-α/IL-6 levels, increased apoptosis, and improved survival (Debnath et al., 2022[13]). These studies progressed from stromal effects to tumor response and targeted TNFR1 disruption. Future priorities include patient stratification based on cytokine signatures and the development of anti-inflammatory modulators for tumor control.

## Pathophysiological Impact of TNFR1-Mediated Necrosis on Breast Cancer Progression

### Promotion of angiogenesis, invasion, and metastatic potential

Necroinflammation associated with TNFR1, when combined with the activation of endothelial cells and matrix remodeling, promotes angiogenesis, invasion, and metastasis, thereby facilitating tumor growth. Sprowl et al. identified that in docetaxel-resistant MCF-7TXT9/10 cells, chemotherapy induced a self-sustaining TNF/NF-κB loop, characterized by increased TNF-alpha production and enhanced NF-kappaB DNA binding. However, this loop can be interrupted by selectively inhibiting TNFR2, which restores the sensitivity of the cells to chemotherapy (Sprowl et al., 2012[60]). Furthermore, Bilecova-Rabajdova et al. observed an upregulation of endothelial DR6 expression at both the transcript and protein levels, which was associated with Ki-67, consistent with pro-angiogenic remodeling of blood vessels (Bilecova-Rabajdova et al., 2014[4]).

Cai et al. (2017[6]) identified that TNF-alpha promotes a pro-metastatic phenotype by upregulating NF-KB and cyclin D1, which enhances cell proliferation, xenograft growth, and microvessel density, which are linked to neovascularization and invasive growth (Cai et al., 2017[6]). He et al. (2021[24]) revealed that TNFR1 activation leads to apoptosis, whereas TNFR2 promotes cell proliferation. Manipulating these pathways can decrease TNF-alpha/IL-6 levels in the tumor microenvironment, thereby inhibiting angiogenesis and tumor invasion (He et al., 2021[24]). Van Berckelaer et al. (2024[62]) discovered that inflammatory breast cancers with high XIAP levels contain CD163 3 tumor-associated macrophages and PD-L1, suggesting niches that support metastasis (Van Berckelaer et al., 2024[62]). Collectively, TNF/NF-KB loops triggered by chemotherapy, endothelial activation and remodeling, inflammation-driven invasion and metastasis, receptor-level bias and cytokine regulation, and immune-suppressive niches demonstrate a connection between necroinflammation and angiogenesis. This supports three strategies: disrupting TNF/TNFR2 feedback during taxane therapy to reduce invasion, integrating vascular-targeted therapies with cytotoxic regimens for DR6-dependent proliferation markers, and preventing TNF/TNFR2 interaction while promoting TNFR1-mediated therapeutic cell death to break pro-angiogenic circuits. 

### Contribution to therapeutic resistance and disease relapse

Inflammatory signaling mediated by TNFR1, which exhibits resistance to therapeutic interventions, promotes cellular survival under cytotoxic stress and maintains residual clones that may contribute to disease recurrence. This condition is sustained by the activation of NF-kB, antiapoptotic mechanisms and cytokine feedback loops. Sprowl et al. demonstrated in breast cancer models treated with taxanes that docetaxel induced TNF-α, and resistant MCF-7TXT9/10 cells exhibited increased TNF-α production with heightened NF-kB binding. Inhibition of TNF-2 restored drug sensitivity, whereas suppression of NF-kB enhanced the effect of docetaxel (Sprowl et al., 2012[60]). This establishes a chemotherapy→TNF→NF-KB loop that stabilizes the survival mechanisms during treatment. Cai et al. found that TNF-alpha promotes breast cancer cell growth, increases tumor burden, and prevents apoptosis through NF-KB targets (Cai et al., 2017[6]). Zhang et al. demonstrated that transmembrane TNF-α confers resistance to doxorubicin by inhibiting caspase-3 and increasing Bcl-2 levels, whereas neutralizing membrane TNF reverses these effects (Zhang et al., 2018[79]). Clinically, He et al. discovered that TNFR1 positivity is associated with higher grade and longer progression-free survival, whereas TNFR2 positivity is correlated with nodal metastasis (He et al., 2021[24]). Van Berckelaer et al. found that tumors with elevated XIAP levels exhibit more nodal involvement and shorter disease-free intervals than those with low XIAP (Van Berckelaer et al., 2024[62]). Zou et al. showed that heregulin reprograms TNFR1 to support survival by reducing apoptosis and increasing Bcl-2 (Zou et al., 2020[82]). These studies indicate that chemotherapy activates TNF signaling, enhancing survival programs; the receptor context influences drug tolerance, while receptor profiles predict progression risk; and growth factors can switch TNFR1 to a survival mode. Therapeutic strategies may include the use of TNF/TNFR2 inhibitors during taxane treatment, combination of NF-kB regulation with cytotoxic drugs, and profiling of TNFR1/TNF2/XIAP to identify high-risk patients.

## Therapeutic Modulation of TNFR1-Linked Necrotic Pathways

### Small-molecule and biologic inhibitors targeting necroptotic mediators

Strategies aimed at targeting necroptosis, encompassing both small-molecule and biologic approaches, are designed to mitigate the effects of TNFR1-mediated cell death and its subsequent effects on angiogenesis, invasion, and immune modulation. These strategies operate by either inhibiting the TNF-alpha/TNFR1 pathway or disrupting the RIPK1-RIPK3-MLKL signaling cascade, thereby limiting vascular adaptability and preventing tumor recurrence. This approach is based on the tumor burden along this pathway. Nicolè et al. (2022[50]) identified a significant increase in RIPK1, RIPK3, and MLKL in aggressive diseases; elevated RIPK3 levels were associated with reduced five-year survival rates; MLKL expression was prevalent in triple-negative tumors; and silencing RIPK1 reduced invasion, highlighting this axis as a potential therapeutic target (Nicolè et al., 2022[50]). Building on this pathological evidence, Liu et al. (2016[41]) observed that necroptotic activity was linked to tumor growth and new blood vessel formation, with increased levels of p-MLKL and RIPK1/3/necroptotic activity, along with higher microvessel density and tumor burden, reinforcing the case for pathway inhibition in the presence of the necroptotic program (Liu et al., 2016[41]).

The endothelium plays a pivotal role in understanding the vascular dynamics. Hänggi et al. (2017[23]) demonstrated that enforced RIPK3 resulted in the destabilization of endothelial barriers across various experimental conditions, whereas inhibiting RIPK1 reduced leakage, and the absence of RIPK3 impeded metastatic spread. This indicates that targeting RIPK1 may prevent both dissemination and intratumoral necroinflammation. This mechanism is associated with ligand-receptor interactions. TNF-alpha promotes growth, activates NF-KB, encourages invasion, and inhibits apoptosis in breast cancer models (Hänggi et al., 2017[23]). However, Cai et al. (2017[6]) disrupted this process, showing that blocking TNF-alpha signaling in response to cytokine overload suppressed proliferation and prevented apoptosis (Cai et al., 2017[6]).

These elements represent interventional data on vascular plasticity that inform treatment strategies. According to Li et al. (2023[38]), RIPK1 activation enhances vasculogenic mimicry, whereas RIPK1 inhibition reduces this characteristic. Tumors exhibiting increased necroptosis also demonstrate denser microvasculature and decreased survival rates (Li et al., 2023[38]). By integrating Nicolè's biomarker data with Liu's insights on growth and angiogenesis, Hänggi's findings on barriers and metastasis, and Cai's cytokine-driven survival program, a coherent strategy emerges: employing RIPK1 prophylaxis and TNF-axis blockade in biomarker-enhanced diseases, using it alone or in conjunction with cytotoxic or immune agents, to reduce permeability, limit vasculogenic mimicry, and slow disease progression in accordance with necroptotic pathways.

### Synergistic strategies integrating necroptosis modulation with standard therapies

Innovative strategies that integrate the regulation of necroptosis with cellular therapies aim to transform TNFR1-mediated cell death into sustained control and limit cell evasion through inflammation. This can theoretically be achieved by combining the effects of TRAIL agonism, chemotherapy, radiotherapy, or checkpoint inhibition with a modulator of the RIPK1 RIPK3/MLKL axis or TNF/TNFR signatures, ensuring a unified outcome for the cells. Foundational evidence for this concept was provided by Chinnaiyan et al. (2000[8]), who demonstrated that combining doxorubicin or paclitaxel with TRAIL resulted in significant apoptosis, characterized by notable caspase-8 activation and cytochrome c release, rather than minimal apoptosis (Chinnaiyan et al., 2000[8]). Addressing the issue of which patients might benefit from such intensified treatment, Nicolè et al. (2022[50]) identified tumors with elevated RIPK1/3/MLKL levels and poorer prognoses, supporting the use of biomarkers to guide the addition of pathway-targeted agents to standard care rather than applying them indiscriminately (Nicolè et al., 2022[50]).

Building on the research conducted by Liu et al. (2016[41]), mechanistic reinforcement has demonstrated that CRISPR-mediated loss of RIPK1/3/MLKL effectively suppresses anchorage-independent growth and the establishment of xenografts. Furthermore, necrostatin-1 has been proven effective in inhibiting tumors characterized by elevated p-MLKL levels, whereas a cytokine-rich environment results in a loss of viability. The absence of ATM increases radiosensitivity, suggesting a strategic combination with radiotherapy when the necroptotic pathway is activated (Liu et al., 2016[41]). In a related study, Cai et al. (2017[6]) found that TNF-alpha facilitates proliferation, colony formation, xenograft growth, and microvascular development via NF-kB. These effects can be mitigated through TNF/TNF receptor (TNFR) antagonism, indicating that survival mechanisms may be neutralized prior to the activation of pro-death pathways (Cai et al., 2017[6]). Additionally, He et al. (2021[24]) identified distinct roles for TNFR1 and TNFR2, demonstrating that the inhibition of TNFR2 alone can impede tumor growth and metastasis. This finding paves the way for future immunotherapy combinations that leverage rather than oppose receptor interactions (He et al., 2021[24]).

**Figure 6:**
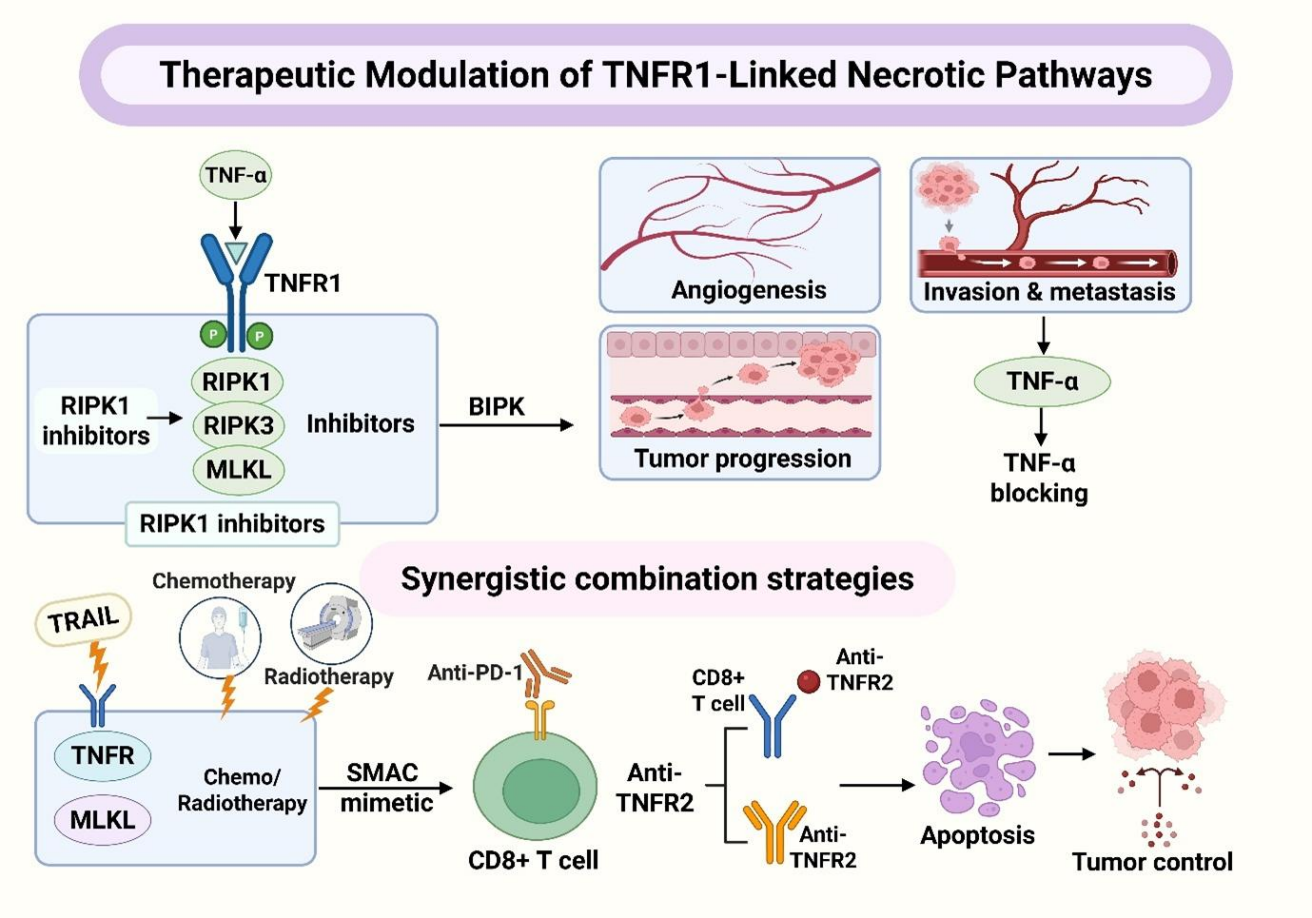
Translational roadmap for TNFR1-directed necroptosis: biomarker panel and rational combination strategies in breast cancer

Fu et al. (2021[19]) implemented this concept in practice and discovered that the combination of anti-TNFR2 and anti-PD-L1 resulted in significant tumor efficacy, enhanced intratumor CD8+ T cells, reduced Tregs, and increased IFN-gamma levels, indicating that receptor-proximal rewiring can unlock checkpoint efficacy. Through a pro-apoptotic pharmacological approach, Yu et al. (2022[76]) demonstrated that a Smac mimetic combined with TRAIL significantly increased apoptosis and reduced xenograft burden compared to TRAIL alone, aligning with the early Chinnaiyan model and offering a feasible template in the context of a neutralized survival pathway (Fu et al., 2021[19], Yu et al., 2022[76]). The vascular invasion axis completes the cycle in triple-negative breast cancer. Li et al. (2023[38]) found that RIPK1 activation promotes vasculogenic mimicry, whereas RIPK1 inhibition eliminates it and is associated with denser but less favorable vascular remodeling and decreased survival, demonstrating that any cytotoxic or immune partner in the presence of vascular plasticity must involve RIPK1 regulation (Li et al., 2023[38]). Furthermore, Liu et al. (2023[42]) showed that Mlkl deletion prevents pulmonary dissemination and reduces soluble adhesion ligands by blocking ADAM10/17, with the antimetastatic effect relying on CD8+ T-cell activity; thus, the ability to assess immune responses to a necroptosis-based strategy is crucial (Liu et al., 2023[42]). Collectively, these findings support biomarker-guided pairing of TRAIL/Smac mimetics, RIPK1/MLKL inhibitors, and TNFR2 or PD-L1 blockade with cytotoxic agents and radiotherapy. Current priorities include optimizing sequencing to avoid cytokine rebound, integrating p-MLKL/TNFR bias as stratifiers, and conducting trials powered by immune and vascular pharmacodynamics alongside survival endpoints (Figure 6(Fig. 6)).

## Conclusion and Future Perspectives

TNFR1-driven necroptosis plays a pivotal role in breast cancer by integrating rapid death signaling with epigenetic reprogramming, thereby establishing a stable therapeutic response. The activation of TNFR1 triggers hierarchical cascades: the formation of Complex I with ubiquitin scaffolding supports NF-kB-driven survival, while the degradation of this scaffold redirects signaling towards the assembly of necrosomes by RIPK1 and RIPK3, culminating in MLKL-dependent membrane permeabilization. Both levels contribute to epigenetic regulation of gene expression. Direct pathways involve TNFR1-mediated recruitment of DNMTs, histone acetyltransferases (such as p300/CBP), and chromatin remodelers (such as SWI/SNF) to promoters, thereby resetting access to survival and death programs in the cell. Indirect mechanisms operate through microRNA networks that target epigenetic enzymes, long non-coding RNAs that scaffold repressive or activating complexes, and metabolic rewiring that alters the availability of cofactors for methylation and demethylation reactions. Concurrently, biomarker development has focused on the abundance of TNFR1, activation states of RIPK1/RIPK3, and epigenetic patterns, including DNA methylation and histone-mark signatures, which can be utilized to stratify for rational intervention. However, there are foreseeable limitations in therapeutic translation. TNF-α signaling has dual effects: necroptosis can trigger antitumor immunity through DAMP release and dendritic cell activation; however, the same inflammatory stimulus can alter stroma structure, enhance angiogenesis, and facilitate metastasis. Outcomes depend on context, subtype heterogeneity, microenvironmental composition, and interpatient variation in chromatin states. Thus, the likelihood of promoting pro-metastatic inflammation necessitates biomarker-guided selection and close safety monitoring. Unanswered questions include when TNFR1 activation inhibits or promotes disease, how to standardize the temporal measurement of epigenetic dynamics in patients, and which chromatin structures predict responsiveness or resistance. The theoretical direction is to focus on combination design, pairing epigenetic modulators (e.g., DNMT and HDAC inhibitors) with TNFR1-pathway intervention, integrate complex epigenetic reads into trial designs, and create safeguards to offset inflammatory toxicity without affecting efficacy. The translational goal is precision therapy, where the combination of epigenetic signatures and TNFR1-pathway status determines targeted therapy and overcomes the signaling outcomes. This will require long-term collaboration between discovery scientists, clinicians, and regulators to certify standardized clinical epigenetic assays, multidimensional data streams, and computational schemes to optimize treatments and deploy adaptive trial designs based on biomarker-defined subgroups. Priority initiatives include the implementation of patient-derived systems with retained native epigenetic and immune properties, reversible epigenetic agents with real-time pharmacodynamics, and regulatory pathways for rational combinations. Combining epigenetic targeting with TNFR1 modulation re-packages therapy as a multi-layer solution that addresses both short-term cell death cues and long-term transcriptional memory, providing a plausible pathway for long-lasting disease control in breast cancer.

## Declaration

### Competing interests 

The authors declare no conflicts of interest. The authors have no financial relationships with the organization that sponsored the research. 

### Author's contribution 

Misbahuddin Rafeeq, Muhammad Afzal: Investigation, Writing-original draft & formal analysis; Muhammad Shahid Nadeem: Visualization; Alaa Hamed Habib: Formal analysis; Hadeel A Alsufyani: Formal analysis & data curation; Sami I. Alzarea: Resources, investigation & conceptualization; Omar Awad Alsaidan: Resources, investigation & conceptualization; Imran Kazmi: Supervision, investigation & conceptualization. 

### Usage of Artifical intelligence 

We declare that AI was not used for the preparation of this manuscript 

### Acknowledgment

This project was funded by the Deanship of Scientific Research (DSR) at King Abdulaziz University, Jeddah, Saudi Arabia under grant no. DRP-1-130-2025. The authors, therefore, acknowledge with thanks DSR for technical and financial support.

## Figures and Tables

**Table 1:**
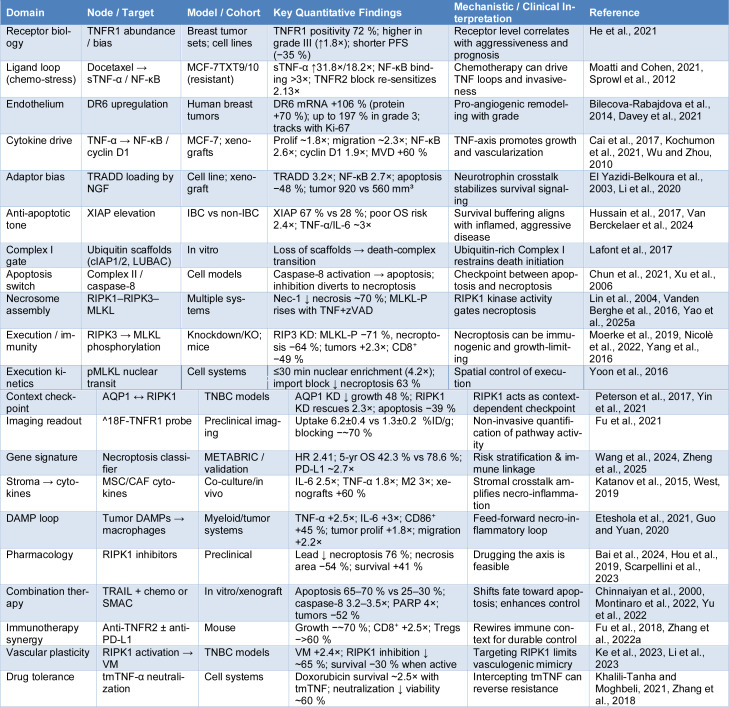
Therapeutic and mechanistic landscape of TNFR1-linked necro-inflammation in breast cancer

**Figure 1:**
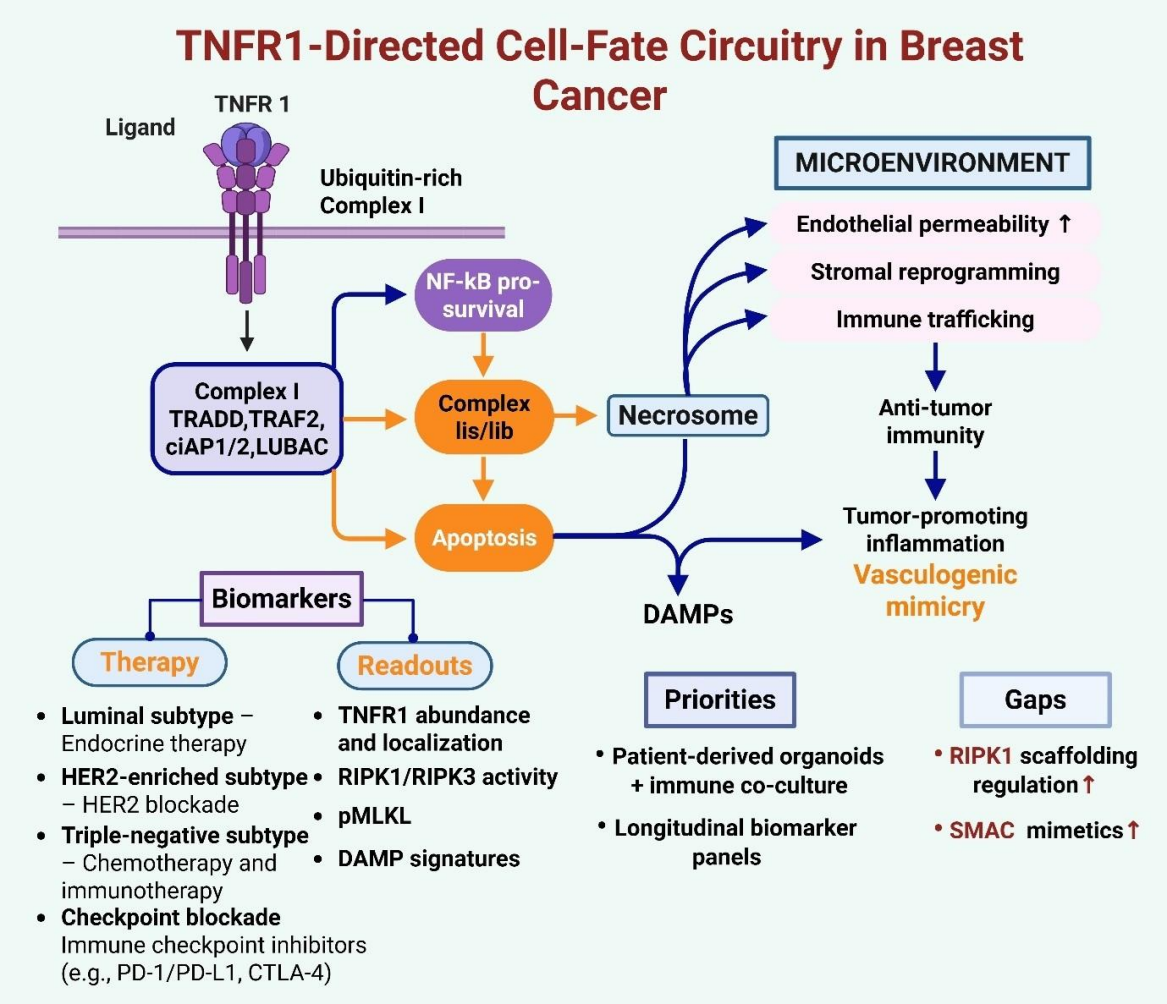
Graphical abstract
